# The Dental and Oral Science Magazine

**Published:** 1878-03

**Authors:** 


### THE DENTAL AND ORAL SCIENCE MAGAZINE.

We have received the first number of this jouanal, published by R. S. Williams, New York. It is well gotten up and is filled with interesting matter. The prospectus says: "This magazine will also be the official organ of the Odontological Society, being the only method of publication which the Society now uses for the presentation of its transactions, and for communication with its members, and with the profession."

We hope the "*Magazine*" will receive the support it merits.

